# Recent Advances in the Isolation Strategies of Plant-Derived Exosomes and Their Therapeutic Applications

**DOI:** 10.3390/cimb47030144

**Published:** 2025-02-22

**Authors:** Jeong-Geon Mun, Dong-Ha Song, Ji-Ye Kee, Yohan Han

**Affiliations:** 1 Department of Oriental Pharmacy, College of Pharmacy, Wonkwang-Oriental Medicines Research Institute, Wonkwang University, 460 Iksandae-ro, Iksan 54538, Jeonbuk, Republic of Korea; wjdrjs05@wku.ac.kr; 2 Department of Microbiology, Wonkwang University School of Medicine, Wonkwang University, 460 Iksandae-ro, Iksan 54538, Jeonbuk, Republic of Korea; sdh12933@wku.ac.kr; 3 Institute of Wonkwang Medical Science, Wonkwang University, 460 Iksandae-ro, Iksan 54538, Jeonbuk, Republic of Korea

**Keywords:** plant-derived exosome-like nanovesicles, drug delivery, ultracentrifuge, density gradient centrifugation, polymer-based precipitation, size-exclusion chromatography

## Abstract

Exosome-like nanovesicles (ELNs) derived from natural products are gaining attention as innovative therapeutic agents due to their biocompatibility, low immunogenicity, and capability to transport bioactive molecules such as proteins, lipids, and nucleic acids. These plant-derived ELNs exhibit structural similarities with mammalian exosomes, making them suitable for drug delivery, microbiome-targeted therapies, and regenerative medicine. Recent studies highlight their potential in treating cancer, inflammation, and metabolic disorders. Additionally, ELNs have applications in cosmetics, agriculture, and the food industry. This review combines the latest advancements in research on plant-derived ELNs, focusing on isolation techniques, pharmacological effects, and therapeutic applications. Although plant-derived ELNs offer promising opportunities, several challenges must be addressed, including standardization, large-scale production, and in vivo efficacy. By summarizing cutting-edge studies and suggesting future directions, we aim to inspire further development of plant-derived ELNs as next-generation therapeutic platforms.

## 1. Introduction

Exosomes are small (30–150 nm in diameter) extracellular vesicles secreted into the extracellular environment by various cell types. Exosomes form within endosomal compartments and are excreted when multivesicular bodies (MVBs) merge with the plasma membrane [1]. Exosomes carry a variety of proteins, lipids, and nucleic acids, which can be delivered to recipient cells, affecting a wide range of physiological and pathological processes. The role of exosomes in mediating intercellular communication has made them important players in numerous biological functions, including immune responses, tissue repair, and the progression of diseases such as cancer and neurodegenerative disorders [2]. The growing interest in exosomes as a research target comes from their potential to serve as informative biomarkers for disease diagnosis and prognosis, given their presence in bodily fluids and their molecular signatures reflective of their cells of origin. Tumor-derived exosomes can carry oncogenic proteins and RNAs, making them valuable for early cancer detection [3]. Similarly, changes in the composition of exosomes can indicate cardiac stress or damage in cardiovascular diseases [4]. Moreover, in neurodegenerative diseases, exosomes in cerebrospinal fluid may contain pathological proteins within exosomes that actively contribute to the progression of Alzheimer’s and Parkinson’s disease [5].

In addition to their diagnostic potential, exosomes offer promising applications in therapeutic delivery systems due to their natural origin, biocompatibility, and ability to deliver therapeutic agents directly to target cells [6]. Exosomes derived from stem cells have been shown to promote tissue repair and regeneration, offering potential treatments for injuries and degenerative diseases [7]. Advances in isolation and characterization techniques, such as nanoparticle tracking analysis, high-throughput sequencing, and advanced microscopy, have further facilitated the detailed study of exosomes [8]. The diverse roles of exosomes in intercellular communication, disease processes, and therapeutic applications are becoming a major focus of contemporary biomedical research, highlighting their potential for revolutionizing healthcare. Recently, many studies have shown great research interest in plant-derived exosomes as well as animal-cell-derived exosomes. One of the most remarkable aspects of plant-derived exosomes is their potential for therapeutic applications. Due to their natural origin, these vesicles are biocompatible and less likely to induce immune reactions than synthetic nanoparticles [9]. Studies have demonstrated that plant-derived exosomes can deliver bioactive compounds to mammalian cells, thereby modulating various cellular processes. In addition, exosomes have attracted significant attention in the field of drug delivery due to their unique properties and ability to overcome various challenges associated with conventional drug delivery systems [10]. Moreover, plant-derived exosomes are being explored for their role in interspecies communication. Emerging evidence suggests that these vesicles can cross species barriers, facilitating the transfer of genetic material and bioactive compounds from plants to animals and even humans. This interspecies communication could have significant implications for understanding the impact of diet on human health and for developing new strategies to harness plant-derived vesicles for therapeutic purposes [11]. Therefore, this review aims to explore and introduce fundamental aspects of plant-derived exosomes, including their isolation methods and pharmacological activities, to contribute to expanding research in this field.

## 2. Characteristics of Plant-Derived Exosomes

Plant-derived exosomes, also known as plant-derived exosome-like nanovesicles (ELNs) or plant-derived lipid nanoparticles, represent a promising area of research within extracellular vesicles. Given that the terms are often used interchangeably in research, we adopt plant-derived exosomes and do not distinguish between these terms in this review [12]. These nanovesicles are secreted by various plant cells and are involved in key physiological processes like plant growth, development, and defense against pathogens. The biogenesis of plant-derived exosomes involves the formation of MVBs within the cell and their subsequent release into the extracellular space, similar to mammalian exosomes. Found in various edible plants, these vesicles have potential uses in human health and nutrition [13]. Plant-derived exosomes exhibit a complex and highly organized structure like those found in mammalian cells. These vesicles are enclosed by a lipid bilayer membrane that provides structural integrity and shields the internal cargo from enzymatic degradation. The lipid composition of plant-derived exosomal membranes is notably rich in cholesterol, sphingolipids, ceramides, and various phospholipids, which confer both stability and functional specificity to these vesicles [9,14,15,16]. Embedded within the exosomal membrane are numerous integral proteins, including tetraspanins (such as CD9, CD63, and CD81), integrins, and major histocompatibility complex (MHC) molecules, which play crucial roles in exosome formation, release, and the targeting of recipient cells. These proteins facilitate the docking and fusion of exosomes with target cells, thereby enabling the transfer of their cargo [17]. Internally, plant-derived exosomes contain diverse bioactive molecules, including proteins such as cytosolic enzymes, heat shock proteins, and cytoskeleton components, as well as signal transduction proteins [18]. Additionally, plant-derived exosomes are rich in various RNAs, including mRNAs, miRNAs, and other small RNAs, which can modulate gene expression and signaling pathways in recipient cells [19].

## 3. Major Types of Exosome Extraction Methods

### 3.1. Ultracentrifugation

Several techniques have been developed for exosome extraction. Ultracentrifugation typically employs centrifugal forces to separate exosomes based on their size and density. The general procedure involves the removal of cells and large debris through lower-speed centrifugation steps, followed by high-speed ultracentrifugation to isolate exosomes. This method has several advantages, including well-established protocols that are widely used in research settings and the ability to process large sample volumes. However, it is labor-intensive, requiring multiple centrifugation steps and specialized equipment, making it time-consuming and expensive. Additionally, ultracentrifugation poses potential risks of exosome damage, such as structural alterations and aggregation, due to the intense centrifugal forces applied during the process [20].

### 3.2. Density Gradient Centrifugation

Density gradient centrifugation is a well-established technique for exosome isolation, recognized for its ability to achieve high-purity fractions by leveraging buoyant density differentials. Like ultracentrifugation, it relies on high-speed centrifugation to sediment exosomes; however, the use of a density gradient medium allows for a more refined separation, reducing contamination from proteins and larger extracellular vesicles. This method is widely employed in exosome research due to its reproducibility and well-documented protocols, making it a reliable approach for minimizing contaminant carryover. Despite its advantages, density gradient centrifugation remains labor-intensive and time-consuming, requiring multiple fractionation steps, meticulous gradient preparation, and precise fraction collection to ensure consistency. Additionally, as with ultracentrifugation, this technique is highly dependent on specialized equipment, increasing operational costs and limiting accessibility for routine or large-scale applications [21].

### 3.3. Polymer-Based Precipitation

Polymer-based precipitation utilizes polymer reagents—most commonly polyethylene glycol (PEG)—to induce exosome aggregation by excluding water molecules, thereby reducing their solubility. Following an incubation period, exosomes can be collected via low-speed centrifugation. Since this method requires only standard centrifugation equipment, it is suitable for processing large sample volumes and offers a cost-effective alternative to ultracentrifugation. However, the co-precipitation of proteins and nucleic acids remains a significant concern, often necessitating additional purification steps to enhance exosome purity [11].

### 3.4. Size-Exclusion Chromatography

Size-exclusion chromatography (SEC) is a gentle and reproducible technique that enables the isolation of exosomes based on size, minimizing structural and functional alterations. Unlike centrifugation-based methods, SEC exerts minimal physical stress on exosomes, thereby preserving their integrity. Additionally, it offers high reproducibility, relatively short processing times, and scalability for moderate sample volumes, making it a viable alternative for exosome purification. Despite these advantages, the SEC has certain limitations. Column capacity constraints may hinder its applicability for large-scale exosome isolation, and co-elution of similarly sized contaminants, such as proteins and other extracellular vesicles, may reduce purification specificity. Furthermore, this requires specialized columns and additional equipment, increasing the overall cost and complexity of implementation [16].

## 4. Isolation and Purification Methods of Plant-Derived Exosomes

Technological advancements have greatly facilitated the isolation and purification of plant-derived exosomes. Techniques such as differential ultracentrifugation, size-exclusion chromatography, and advanced microscopy are employed to study exosome size, composition, and functional properties [22]. These processes ensure the acquisition of high-purity vesicles necessary for reliable experimental results and potential therapeutic applications. Isolation and purification are critical steps in studying the structure, composition, and functional properties of plant-derived exosomes. Each technique for these purposes offers distinct advantages and limitations, contributing to the overall understanding and utilization of exosomes.

### 4.1. Ultracentrifugation

Differential ultracentrifugation is one of the most commonly used methods for isolating plant-derived exosomes. This technique involves a series of centrifugation steps at increasing speeds to separate exosomes from other cellular debris and larger vesicles. Initially, low-speed centrifugation removes cell debris and larger particles, followed by high-speed centrifugation to pellet the exosomes. While differential ultracentrifugation is widely used due to its simplicity and cost-effectiveness, it can result in contamination from other vesicles and protein aggregates, potentially affecting the purity of the exosomal preparation [23]. The plant-derived exosomes isolated by ultracentrifuge are listed in Table 1, and more details are illustrated in Section 4.

### 4.2. Density Gradient Centrifugation

Density gradient centrifugation is another technique that improves the purity of isolated exosomes. This method uses a density gradient medium such as sucrose or iodixanol during ultracentrifugation. While sucrose gradients have been widely used for years, iodixanol is increasingly preferred due to its ability to preserve exosome biological activity better. It has been reported that sucrose may inhibit the biological effects of exosomes, whereas iodixanol maintains its structural integrity under iso-osmotic conditions. Additionally, iodixanol is metabolically inert and has a lower viscosity, making it advantageous for exosome isolation [33]. Although Li et al. [34] used iodixanol to isolate exosomes from ginger rhizomes, our review indicates that most researchers working with plant-derived exosomes still prefer sucrose-based density gradient centrifugation. Exosomes migrate to their respective buoyant densities within the gradient, allowing for a more refined separation from other vesicular contaminants. This method is more effective in achieving high-purity exosomal fractions than standard differential ultracentrifugation, but it is more time-consuming and requires precise handling. Additionally, this technique cannot differentiate exosomes from other vesicles like viral particles or microvesicles due to their similar buoyant densities [35]. The plant-derived exosomes isolated by density gradient centrifugation are listed in Table 2, and more details are illustrated in Section 4.

### 4.3. Polymer-Based Precipitation

Polymer-based precipitation is another method where water-excluding polymers, such as polyethylene glycol (PEG), are added to the sample to precipitate exosomes. This relatively simple and scalable technique makes it suitable for large-scale exosome production. However, polymer-based precipitation can co-precipitate other extracellular components, potentially reducing the purity of the isolated exosomes [11]. The plant-derived exosomes isolated by PEG are listed in Table 3, and more details are illustrated in Section 4.

### 4.4. Size-Exclusion Chromatography

Size-exclusion chromatography offers an alternative approach for exosome purification based on their size. In size-exclusion chromatography, a column filled with porous phase separates particles as they pass through. Larger particles elute first, followed by smaller ones, allowing the isolation of exosomes in a size-dependent manner. Size-exclusion chromatography is advantageous because it preserves the integrity and functionality of exosomes, minimizing damage from large centrifugal forces. Moreover, size-exclusion chromatography, often combined with other vesicle separation techniques, enhances the efficiency of exosome isolation and purification and can be applied to their morphological and structural analyses. However, size-exclusion chromatography might not eliminate protein contaminants of a similar size to exosomes [16,66]. The plant-derived exosomes isolated by size-exclusion chromatography are listed in Table 4, and more details are illustrated in Section 4.

## 5. Isolation Methods and Therapeutic Potential of Exosomes from Key Plant Species

This section will specifically illustrate exosomes derived from natural products such as medicinal plants and herbal extracts based on their isolation methods. Plant-derived exosomes have attracted much research interest due to their unique bioactive components, including phytochemicals, proteins, and nucleic acids, which can contribute to enhanced therapeutic effects. We will explore the various extraction and purification methods adapted for isolating plant-derived exosomes and discuss how these exosomes can be optimized for disease prevention and treatment. The potential pharmacological activities of plant-derived exosomes, such as anti-inflammatory, anti-oxidant, and anti-cancer effects, will be addressed, suggesting a new concept of natural products to be utilized as innovative nanomedicines. To systematically analyze relevant studies, we conducted a literature search using PubMed for research articles published in the last three years. The search terms included “plant-derived extracellular vesicles,” “plant-derived exosomes,” and “plant-derived nanoparticle.” The inclusion criteria were studies that clearly described plant-derived exosome extraction and isolation methods. Studies lacking sufficient detail on extraction and isolation methods or primarily focusing on plant-derived exosomes as drug delivery carriers rather than their therapeutic properties were excluded.

### 5.1. Catharanthus roseus L.

To isolate Don-leaf-derived exosome, fresh leaves were digested with cellulase (3 g/100 mL) and pectinase (0.2 g/100 mL) for 12 h and concentrated using a hollow fiber module after centrifuging to obtain pure supernatant. Finally, exosomes were isolated using sucrose-buffered ultracentrifugation. Ou et al. [36] found that exosomes isolated from *Catharanthus roseus* L. alleviated cyclophosphamide-induced immunosuppression and strongly stimulated the secretion of TNF-α in vitro and in vivo.

### 5.2. Solanum nigrum L.

Fresh black *Solanum nigrum* L. was ground using a food chopper and filtered using a nylon filter. Then, large particles and cell debris were removed by sequential centrifugation. The supernatant was mixed with PEG 6000 and incubated at 4 °C overnight. The incubated mixture was centrifuged at 8000× *g* for 30 min to precipitate exosomes. The centrifuged pellets were resuspended in ddH_2_O and filtered by a syringe filter with pore sizes of 0.45 μm and 0.22 μm sequentially. Exosomes from *Solanum nigrum* L. exerted an anti-inflammatory effect in LPS-stimulated RAW 264.7 cells by suppressing the expression of pro-inflammatory cytokines [55].

### 5.3. Pueraria lobata

Zhang et al. [37] isolated exosomes from *Pueraria lobata*. The crushed root of *Pueraria lobata* was mixed with PBS by a grinding machine and incubated overnight at 4 °C. After the overnight incubation, the mixture was filtered with medical gauze, and large particles were eliminated by centrifugation. The resulting supernatant was filtered with a 0.22 µm filter and centrifuged at 150,000× *g* for 2 h. To obtain pure exosomes, the pellets were resuspended in PBS and transferred into a sucrose gradient with ultracentrifugation at 150,000× *g* for 2 h. Exosomes from *Pueraria lobata* prevented alcoholic liver injury. Zhan et al. [24] also isolated exosomes from the fresh root of *Pueraria lobata*. Unlike Zhang’s research group [37], who isolated exosomes in a cold environment, they isolated exosomes in double steam water at 60 °C overnight. The extract was filtered with medical gauze and then centrifuged, followed by a 0.45 μm filter. The filtered liquid was ultracentrifuged at 100,000× *g* for 70 min twice. In their study, isolated exosomes exerted a therapeutic function on osteoporosis.

### 5.4. Panax ginseng

Several research groups isolated exosomes from ginseng using various methods. Tan et al. [70] isolated exosomes from ginseng roots. The ginseng was washed and blended to produce juice, which was serially centrifuged to remove debris. The supernatant was then ultracentrifuged at 150,000× *g* for 2 h at 4 °C to pellet the exosomes. The pellets were resuspended in PBS and filtered through a 0.22 μm filter. The purified GExos are safe and effective carriers for diabetic ulcers by modulating glycolysis reprogramming and angiogenesis. Differing from Tan’s group, Yang et al. [38] isolate exosomes from fresh ginseng using density gradient centrifugation methods. The ginseng was thoroughly cleaned, cut, and homogenized with cold PBS. The filtrated supernatant was supplemented with protease inhibitors. The supernatant was then centrifuged to remove large debris, and the resulting supernatant was subjected to ultracentrifugation at 100,000× *g* for 1 h to pellet the exosomes. The exosome pellets were resuspended in 20 mM Tris–HCl and further purified through sucrose density gradient centrifugation at 150,000× *g* for 3 h at 4 °C. The intermediate bands containing purified ginseng-derived exosomes were collected. In this study, the exosomes ameliorate inflammatory bowel disease induced by DSS via the TLR4/MAPK and p62/Nrf2/Keap1 pathways. Using density gradient centrifugation methods, Kim et al. [30] also isolated exosomes from four-year-old ginsengs. They were thoroughly washed and ground in 30 mL of PBS using a blender. The resulting ginseng juice was filtered through a sieve to remove coarse debris. The filtered juice was sequentially centrifuged to remove residual large particles. The resulting supernatant was then filtered through a 0.2 μm syringe filter. This filtrate was layered onto sucrose cushions and ultracentrifuged at 100,000× *g* for 90 min. The sucrose cushion was further purified using a sucrose gradient at 200,000× *g* for 90 min, isolating exosomes from the 8–30% interface. The isolated exosomes exert therapeutic effects on glioma both in vitro and in vivo, demonstrating the potential for inhibiting glioma progression and modulating tumor-associated macrophages. Interestingly, Choi et al. [25] isolated exosomes from air-dried ginseng root, not fresh ginseng. Air-dried ginseng root was ground using a blender. The juice was soaked, and exosomes were isolated through serial ultracentrifugation at 150,000× *g*, 4 °C for 70 min. The exosomes protect against skin damage caused by UV and oxidative stress.

### 5.5. Portulaca oleracea L.

The *Portulaca oleracea* L. was washed and homogenized in PBS to extract the juice, which was processed through sequential centrifugation steps. Serial centrifugation was used to pellet exosomes to eliminate large particles. The pellets were resuspended in PBS and purified through a sucrose gradient with ultracentrifugation. The isolated exosomes were resuspended in PBS, and these exosomes ameliorated DSS-induced colitis by expanding double-positive CD4^+^CD8^+^T cells [40].

### 5.6. Pachyrhizus erosus (Yam Bean)

To isolate *Pachyrhizus erosus*-derived exosomes, yam bean tubers were ground in a juicer. The juice was filtered to remove large particles and cell debris. The filtered juice was sequentially centrifuged, and the final supernatant was mixed with 12% PEG 6000 at a 1:1 ratio (*v*/*v*) overnight. It was then centrifuged at 10,000× *g* for 1 h to precipitate exosomes. The pellets were resuspended in aqua bidest and filtered through syringe filters (0.45 μm and 0.22 μm). The extracted exosomes exert an anti-melanogenic effect in skin treatment [56].

### 5.7. Ginger Rhizomes

Kalarikkal et al. [57] isolated exosomes using modified conventional protocols. Fresh ginger rhizomes were ground, and the resulting juice was filtered through a nylon mesh (125 μm). The filtered juice was sequentially centrifuged to remove large particles, yielding the S10 extract. This extract was used for exosome purification, and two methods were used: First, ultracentrifugation was performed at 125,000× *g* for 2 h at 4 °C. Second, PEG was used. The S10 extract was mixed with PEG overnight at 4 °C. After centrifugation at 8000× *g* for 30 min, excess PEG was removed by inverting the tubes on tissue paper. The resulting exosome pellets from both methods were resuspended in sterile water and lyophilized. The isolated exosomes exhibit anti-oxidant activity in hydrogen-peroxide-treated murine macrophages.

### 5.8. Carica papaya L.

Iriawati et al. [58] isolated exosomes using a PEG 6000. Raw and ripe papaya were ground into juice and filtered through nylon meshes (100 μm and 40 μm). The filtrate was treated with pectolyase to digest pectin enriched in juice. The filtrate was mixed with aqua bidest and sequentially centrifuged to remove large debris. The supernatant was mixed with PEG 6000 overnight and centrifuged at 8000× *g* for 30 min. The pellets were resuspended in aqua bidest with 25 mM trehalose and filtered through 0.45 μm and 0.22 μm filters. Isolated exosomes exerted an anti-inflammatory effect in LPS-induced RAW 264.7 cells by inhibiting pro-inflammatory cytokine and NO production. In addition, PDENs inhibited cell migration of macrophages and neutrophils in the zebrafish model.

### 5.9. Physalis minima (Golden Cherry)

Setiadi et al. [59] isolated exosomes from golden cherry. Golden cherry was blended and filtered (0.22 μm) to remove large debris. The filtrate was sequentially centrifuged, and the resulting pellets were mixed with PEG 6000 to achieve a 10% final concentration and centrifuged at 8000× *g* for 30 min to isolate the exosomes. The isolated exosomes have been successfully incorporated into gel formulations, demonstrating potential as a novel therapeutic approach for the treatment of photoaging.

### 5.10. Mulberry Bark

Sriwastva et al. [41] isolated exosomes by manually removing and blending mulberry bark tissues. The slurry was filtered through a strainer, and the filtrate was differentially centrifuged to remove large debris. Then, the supernatant was ultracentrifuged with a sucrose density gradient, followed by ultracentrifugation at 150,000× *g* for 2 h. The isolated exosomes prevented DSS-induced colitis via the AhR-COPS8-mediated anti-inflammatory pathway.

### 5.11. Allium sativum (Garlic)

Sundaram et al. [42,43] isolated *Allium sativum* exosomes by blending peeled *Allium sativum* and filtering the juice to remove large particles. The filtrate was sequentially centrifuged to remove microparticles and ultracentrifuged at 150,000× *g* for 2 h. Exosomes were purified using a sucrose gradient and ultracentrifugation at 150,000×g for 2 h. Isolated exosomes regulate high-fat-diet-induced weight gain and type 2 diabetes through regulation of the gut/brain axis. Wang et al. [44] also isolated *Allium sativum* exosomes from peeled *Allium sativum*. It was homogenized, and the juice was filtered to remove large fibers. Then, the supernatant was sequentially centrifuged, and exosomes were pelleted using ultracentrifugation. The pellets were repurified using a sucrose gradient and ultracentrifuged. The 30%/45% fraction was collected and washed with PBS to remove sucrose. Notably, exosomes treated DSS-induced colitis by reshaping gut microbiota.

### 5.12. Vitis vinifera (Grape)

Ju et al. [45] isolated exosomes from peeled *Vitis vinifera*. It was pressed in a cold room to extract juice and was diluted with cold PBS. The mixture was differentially centrifuged and purified by a sucrose gradient to isolate exosomes. The sucrose-purified exosomes ameliorate DSS-induced colitis by targeting intestinal stem cells.

### 5.13. Allium tuberosum

Ishida et al. [60] isolated exosomes from *Allium tuberosum* using differential centrifugation. Fresh *Allium tuberosum* was crushed in water and then filtered. The extract was differentially centrifuged, and the resulting supernatant was further filtered through a 0.8 μm filter. Exosomes were subsequently extracted using the exoEasy Maxi Kit (QIAGEN, Hilden, Germany). Isolated exosomes alleviate neuroinflammation in microglia-like cells.

### 5.14. Brassica oleracea L. (Broccoli)

Wang et al. [26] isolated exosomes from fresh *Brassica oleracea* L. by ultracentrifugation. First, the *Brassica oleracea* L. was blended using a blender. The resulting juice was sequentially centrifuged to remove large debris. After that, the supernatant was subjected to ultracentrifugation to collect the exosomes. These exosomes induce apoptosis in human pancreatic cancer cells by targeting IRS1. Differing from Wang’s research group, Duan et al. [46] isolated exosomes through density gradient centrifugation. They homogenized *Brassica oleracea* L. using a blender, and the juice was centrifuged sequentially to remove large particles. The supernatant was ultracentrifuged and purified using a sucrose gradient. Exosomes from sucrose layers were ultracentrifuged again to remove sucrose. Isolated exosomes relieve loperamide-induced constipation by regulating gut microbiota and tryptophan metabolism.

### 5.15. Houttuynia cordata

Zhu et al. [27] isolated *Houttuynia cordata*-derived exosomes. *Houttuynia cordata* was blended, and the resulting juice was sequentially centrifuged to remove large debris. After that, the supernatant was ultracentrifuged to collect the exosomes. These exosomes exert an antiviral effect against the respiratory syncytial virus.

### 5.16. Atractylodes lancea Rhizome

Differing from other plant-derived exosomes, *Atractylodes lancea* rhizome-derived exosomes were isolated from hot water extract. Ishida et al. [61] isolated exosomes from dried rhizomes of *Atractylodes lancea*. The hot water extract was sequentially centrifuged, and the final supernatant was filtered through a 0.8 μm filter. The exosomes were isolated from the filtrate using the exoEasy Maxi Kit. Isolated exosomes inhibit melanogenesis in B16-F10 melanoma cells. Kawada et al. [62] also isolated exosomes from *Atractylodes lancea* rhizomes. The *Atractylodes lancea* rhizomes were boiled, and the filtered extract was centrifuged sequentially. The supernatant was filtered through a 0.8 μm filter, and the exosomes were extracted using the exoEasy Maxi Kit. Isolated exosomes showed an anti-inflammatory effect in LPS-treated murine microglial cells.

### 5.17. Citrus limon

Raimondo et al. [47] isolate exosomes from manually squeezed *Citrus limon* L. juice. The filtered juice was sequentially centrifuged, and the supernatant was ultracentrifuged. A 30% sucrose/D_2_O cushion repurified the pellets. These exosomes suppress chronic myeloid leukemia (CML) tumor growth in vivo by activating TRAIL-mediated apoptosis.

### 5.18. Salvia miltiorrhiza

Zhang et al. [48] isolated exosomes from fresh *Salvia miltiorrhiza*. The washed plants were homogenized in PBS and filtered to remove large particles. The filtrate was sequentially centrifuged, and the resulting precipitate was ultrasonicated in an ice-water bath for 25 min. The suspension was further purified by sucrose density gradient with ultracentrifugation. These exosomes promote angiogenesis by enhancing cell viability and migration in human umbilical vein endothelial cells and improving neovascularization in myocardial ischemia–reperfusion injury, suggesting potential for treating heart tissue damage.

### 5.19. Taraxacum officinale

Zhang et al. [28] isolated exosomes from fresh *Taraxacum officinale*. The washed *Taraxacum officinale* was sequentially centrifuged, and the pellets were ultracentrifuged. Isolated exosomes alleviate intermittent hypoxia-induced hypertension by regulating gut metabolites.

### 5.20. Vaccinium ashei (Blueberry)

Robertis et al. [67] isolated exosomes from blueberry juice. The juice was diluted in PBS and sequentially centrifuged to remove large particles. The supernatant was filtered with a 10,000 MWCO membrane (Sartorius, Göttingen, Germany) and then ultracentrifuged. The exosomes were freshly rinsed with sterile PBS. Notably, isolated exosomes counter the effects of TNF-α-induced gene expression changes in EA.hy926 cells. Zhao et al. [63] also isolated exosomes with an electric blender for juice extraction. The filtered juice was sequentially centrifuged to remove large debris. The supernatant was then incubated with 8% PEG 8000 and centrifuged at 10,000× *g* for 30 min to isolate exosomes. The resulting exosomes alleviate nonalcoholic fatty liver disease by reducing mitochondrial oxidative stress.

### 5.21. Asparagus cochinchinensis

Zhang et al. [49] isolated *Asparagus cochinchinensis*-derived exosomes. *Asparagus cochinchinensis* was squeezed for fresh juice and was differentially centrifuged to remove large particles. The ultracentrifuged supernatant was transferred to a sucrose gradient solution and ultracentrifuged again. The exosomes were collected from the 30%/45% sucrose interface, and the resulting pellets were dried under a nitrogen atmosphere for quantification. The isolated exosomes exhibit cytotoxicity against human HCC cell lines, including HepG2, Hep 3B, and SMMC-7721.

### 5.22. Momordica charantia

Cai et al. [50] isolated *Momordica charantia*-derived exosomes. Fresh *Momordica charantia* was ground, and the juice was centrifuged differentially. The supernatant was ultracentrifuged, and the resuspended pellets were purified using sucrose gradient ultracentrifugation. Isolated exosomes show neuroprotective effects against ischemic brain injury (IBI).

### 5.23. Lycium barbarum L. (Goji Berry)

Zhou et al. [51] isolated goji-berry-derived exosomes. Fresh goji berries were blended and sequentially centrifuged to remove large debris. The ultracentrifuged supernatant was transferred to a sucrose gradient and ultracentrifuged again. The final pellets were filtered through 0.44 μm and 0.22 μm filters. These exosomes ameliorate dexamethasone-induced muscle atrophy by regulating the AMPK/SIRT1/PGC1α signaling pathway.

### 5.24. Citrus limon (Lemon)

Lei et al. [52] isolated *Citrus limon*-derived exosomes. The peeled *Citrus limon* was squeezed and sequentially centrifuged. The pellets from the 36,000× *g* centrifugation step were resuspended and further purified by sucrose gradient ultracentrifugation. The isolated exosomes improve bile resistance in *Lactobacillus rhamnosus* GG by reducing Msp1 and Msp3 levels through tRNA decay.

### 5.25. Platycodon grandiflorum

Kim et al. [68] isolated *Platycodon grandiflorum*-derived exosomes. *Platycodon grandiflorum* was ground in a blender, and the filtered juice was serially centrifuged to remove large debris. The exosomes were isolated from the supernatant using a tangential flow filtration (TFF) system with a 500 kDa TFF membrane (Pall Biotech, Dreieich, Germany). The isolated exosomes loaded with silymarin alleviate acetaminophen-induced hepatotoxicity.

### 5.26. Centella asiatica

Huang et al. [29] isolated *Centella asiatica*-derived exosomes. The leaves of fresh *Centella asiatica* were thoroughly washed and crushed to obtain juice. The juice was sequentially centrifuged, and the filtered final supernatant was ultracentrifuged. The resulting pellets were resuspended in PBS and inhibited the proliferation of HepG2 cells, suggesting the potential utility of exosomes as anticancer agents.

### 5.27. Physalis peruviana (Goldenberry)

Natania et al. [64] isolated exosomes from the goldenberry. The berries were blended for juice. The filtered juice was centrifuged sequentially, and the supernatant was mixed with PEG 6000 overnight at 4 °C. The following day, the mixture was centrifuged again, and the resuspended exosomes were filtered using a 0.22 μm syringe filter. The isolated exosomes are used for human dermal fibroblast regeneration and remodeling. Vanessa et al. [65] also isolated exosomes from goldenberries. Goldenberries were ground, and the juice was centrifuged sequentially. The supernatant was mixed with 12% PEG 6000 overnight at 4 °C and centrifuged. The final pellets were resuspended and filtered through a 0.22 μm syringe filter. The isolated exosomes exert anti-inflammatory potential by regulating macrophage M1/M2 polarization.

### 5.28. Panax notoginseng

Li et al. [53] isolated exosomes from *Panax notoginseng*. Fresh *Panax notoginseng* root was ground and filtered to remove large debris. The filtrate was serially centrifuged, and the supernatant was layered onto a glucose/sucrose cushion and subjected to ultracentrifugation. A sucrose density gradient with ultracentrifugation repurified the layer from the band over the sucrose cushion. The isolated exosomes are effective against ischemia–reperfusion injury by altering microglia polarization.

### 5.29. Brucea javanica

Yan et al. [30] isolated exosomes from *Brucea javanica* juice. The juice was sequentially centrifuged to remove large fibers. The supernatant was ultracentrifuged, and the pellets were filtered through a 0.22 μm membrane. Isolated exosomes were effective for triple-negative breast cancer (TNBC) by delivering functional miRNAs that regulate the PI3K/Akt/mTOR pathway and promote ROS/caspase-mediated apoptosis.

### 5.30. Sesamum indicum L. (Sesame Leaves)

Jiang et al. [69] isolated exosomes from sesame leaves. The sesame leaves were homogenized and centrifuged sequentially to obtain the supernatant. The supernatant was then concentrated using a 100 kDa ultrafiltration membrane and further purified through an ultrapure column (SuperEV5.0) with PBS elution. Isolated exosomes exerted an anti-inflammatory effect in LPS-treated RAW 264.7 cells. In particular, they loaded luteolin to elevate the anti-inflammatory effect of sesame-deprived exosomes.

### 5.31. Citrus paradisi (Grapefruit)

Castelli et al. [31] isolated exosomes from *Citrus paradisi* fruit. The filtered fruit juices were serially centrifuged to remove cell debris and microvesicles. The supernatant was ultracentrifuged at 110,000× *g* for 1.5 h to collect exosomes. Isolated exosomes selectively inhibit leukemic cell proliferation by increasing ROS levels, suggesting potential as a supportive leukemia therapy.

### 5.32. Fragaria × ananassa (Strawberry)

Perut et al. [32] isolated exosomes from *Fragaria* × *ananassa*. The *Fragaria* × *ananassa* juice was differentially centrifuged, and the final supernatant was filtered with a 0.45 µm pore-size filter and ultracentrifuged. The isolated exosomes ameliorated oxidative stress in human mesenchymal stromal cells.

### 5.33. Lycium barbarum L.

Wang et al. [54] isolated exosomes from *Lycium barbarum*. Fresh *Lycium barbarum* was homogenized in PBS, a protease inhibitor, and 1 M Tris–HCl. The homogenate was centrifuged sequentially, followed by ultracentrifugation. The precipitate was resuspended and subjected to a sucrose density gradient with ultracentrifugation. The 30–45% intermediate layer was collected and ultracentrifuged again. The isolated exosomes promote spinal cord injury repair. In particular, they loaded isoliquiritigenin into *Lycium barbarum*-derived exosomes and elevated therapeutic function.

## 6. Conclusions and Future Directions

With the global demand for sustainable, safe, and effective therapeutics, plant-derived exosomes are gaining attention as promising next-generation therapeutics. These naturally generated exosomes possess the potential to be a new drug concept and offer innovative applications across industries, including cosmetics, agriculture, and food science. Therefore, we summarized the diverse applications of plant-derived exosomes, including their isolation processes and potential as therapeutic agents for various diseases. By highlighting exosomes derived from various natural products, we emphasized the potential of plant-derived exosome therapeutics and their broader applications. Plant-derived exosomes, rich in anti-oxidants, vitamins, and lipids, offer significant potential in cosmetics, not only for therapeutic applications. Exosomes from ginseng and green tea rejuvenate skin by regulating oxidative stress, skin hydration, elasticity, and skin hydration [71]. Their nano-size and high permeability enable deep delivery of active ingredients, advancing anti-aging and skin repair therapies. These multifunctional applications highlight plant-derived exosomes as innovative solutions in diverse healthcare.

However, several factors must be thoroughly investigated before these promising plant-derived exosomes are clinically applied. The safety of plant-derived exosomes is essential for their translational potential. While current studies indicate that plant-derived exosomes have low immunogenicity and minimal cytotoxicity in preclinical models, largely due to their natural origin and biocompatible composition, they also possess unique biological properties and functions despite sharing common traits like shape and size. Notably, their absorption time, biodistribution, and pharmacological effects can vary significantly depending on their source. Using the same protocol, Lu et al. [72] isolated exosomes from *Citrus limon*, ginger rhizomes, *Vitis vinifera*, and celery. Interestingly, celery-derived exosomes exhibited superior cellular uptake compared to exosomes isolated from ginger rhizomes, *Vitis vinifera*, and *Citrus limon*. These findings indicate that similar to cell-derived exosomes, plant-derived exosomes can exhibit ununified biological characteristics, including differences in cellular uptake efficiency based on their isolation sources. Furthermore, although exosomes were extracted from various plants known for their anti-oxidant effects, only the exosomes derived from blueberries exhibited anti-oxidant activity [63]. This result shows that the effects of exosomes may not align with the characteristics of their source plants. A significant challenge in standardizing the production of plant-derived exosomes is ensuring the consistency of their quality and characteristics. Variability among plant species plays a crucial role, but cultivation conditions and the specific tissue types used also substantially impact the properties of exosomes. Research by Viršilė et al. [73] shows that different plant environmental cultivation parameters can affect both the yield and bioactivity of exosomes, as demonstrated with exosomes derived from tomato leaves. These findings emphasize the importance of standardizing growth conditions to achieve reproducible quality in exosomes for therapeutic applications.

Additionally, the biodistribution and clearance of plant-derived exosomes in vivo require further investigation. While many studies have demonstrated their rapid uptake by specific tissues or cells, long-term effects and accumulation must be addressed to ensure safety. Zhang et al. [49] isolated exosomes from *Asparagus cochinchinensis*. In vivo distribution studies showed that these exosomes accumulated in healthy and tumor-bearing mice increased after scavenger receptor (SR) blockade. Also, PEGylation of vesicles further enhanced their blood circulation time and tumor site accumulation. Therefore, rigorous characterization of plant-derived exosome composition, including proteins, lipids, and RNAs, is vital to avoid unintended bioactivity. As research progresses, integrating good manufacturing practices for plant-derived exosome production will ensure consistent quality and safety.

Nonetheless, rather than being used solely as therapeutics, these nanoparticles can also serve as drug delivery carriers, suggesting a promising future for the clinical applications of plant-derived exosomes. You et al. loaded DNA oligonucleotides and miRNA into nanovesicles from cabbage [74]. In particular, Umezu et al. [75] encapsulated miRNA in acerola-juice-derived exosomes and administered this engineered vesicle orally. The findings highlight the potential of plant-derived exosomes as an effective oral delivery system for nucleic acids capable of modulating gene expression in the digestive system and liver. This study is significant because it demonstrates that plant-derived exosomes can be used for oral delivery, providing a noninvasive alternative to injections. This could simplify treatments for patients and expand the application of nucleic-acid-based therapies.

Plant-derived exosomes offer a highly promising platform for drug delivery due to their biocompatibility, stability, and naturally occurring bioactive compounds. Unlike synthetic nanocarriers, plant-derived exosomes are derived from natural sources, making them biodegradable and less toxic, thereby enhancing therapeutic outcomes. In addition, emerging research has shown that plant-derived exosomes can cross biological barriers, such as the blood–brain barrier, highlighting their potential in addressing neurological disorders. Furthermore, plant-derived exosomes can be engineered with surface modifications or loaded with specific drugs to improve their efficacy in treating cancers, inflammatory conditions, and metabolic diseases. Their unique ability to interact with gut microbiota also could develop microbiome-targeted therapies. In conclusion, plant-derived exosomes could be a promising platform for future therapeutics, potentially addressing a wide range of medical challenges.

## Figures and Tables

**Table 1:** Exosomes isolated by ultracentrifugation.

Exosome Origin	Extraction Method	Isolation Method	Application	Reference
*Pueraria lobata*	Double steam water at 60 °C	Ultracentrifugation	Osteoporosis (0–20 µM in BMSCs and 10 mg/kg in SD rats)	[24]
*Panax ginseng*	Grinding machine	Ultracentrifugation	Oxidative stress (0–2 × 10^9^ particles/mL in HaCaT cells)	[25]
*Brassica oleracea* L. (broccoli)	Grinding machine	Ultracentrifugation	Apoptosis in human pancreatic cancer cells (0–40 mg/µL in PANC-1 cells)	[26]
*Houttuynia cordata*	Grinding machine	Ultracentrifugation	Anti-viral effect against respiratory syncytial virus	[27]
*Taraxacum officinale*	Not provided	Ultracentrifugation	Intermittent hypoxia-induced hypertension (Protein concentration of 0.5 mg/kg in SD rats)	[28]
*Centella asiatica*	Crush	Ultracentrifugation	Anti-proliferation effect for HepG2 cells (0–80 µg/mL in HepG2 cells)	[29]
*Brucea javanica*	Not provided	Ultracentrifugation	Triple-negative breast cancer (TNBC) (0–200 µg/mL in 4T1, MCF-7 and MDA-MB-231 cells, and 6 mg protein/kg in BALB/c mice)	[30]
*Citrus paradisi* (grapefruit)	Not provided	Ultracentrifugation	Leukemia (0–1 × 10^12^/mL in leukemic and leukemic blast cell lines)	[31]
*Fragaria* × *ananassa* (strawberry)	Squeeze	Ultracentrifugation	Anti-oxidant effect (0–9 µg/mL in human ADMSCs)	[32]

**Table 2:** Exosomes isolated by density gradient centrifugation.

Exosome Origin	Extraction Method	Isolation Method	Application	Reference
*Catharanthus roseus* L.	Digested with cellulase and pectinase	Sucrose density gradient with ultracentrifugation	Immunostimulatory effects (0–240 µg/mL in RAW 264.7 cells and 60 mg/kg in BALB/c mice)	[36]
*Pueraria lobata*	Grinding machine	Sucrose density gradient with ultracentrifugation	Alcoholic liver injury (0–50 mg/kg in C57BL/6J mice)	[37]
*Panax ginseng*	Cut into small pieces	Sucrose density gradient with ultracentrifugation	Inflammatory bowel disease (IBD) (0–20 µg/mL in RAW 264.7 and Caco-2 cells and 0–10 mg/mL in C57BL/6J mice)	[38]
	Grinding machine	Sucrose density gradient with ultracentrifugation	Glioma (0–62.5 µg/mL in C6 glioma cells, 2 mg/1 mL in Wister rats, and 2 mg/5 µL in BALB/c mice)	[39]
*Portulaca oleracea* L.	Grinding machine	Sucrose density gradient with ultracentrifugation	DSS-induced colitis (0–100 mg/g in C57BL/6 mice)	[40]
Mulberry bark	Grinding machine	Sucrose density gradient with ultracentrifugation	DSS-induced colitis (1 × 10^10^ particles/100 µL/mouse in C57BL/6J mice)	[41]
*Allium sativum* (garlic)	Grinding machine	Sucrose density gradient with ultracentrifugation	High-fat-diet-induced weight gain (1 × 10^10^ particles in C57BL/6J mice)	[42]
	Grinding machine	Sucrose density gradient with ultracentrifugation	Type 2 diabetes (High-fat-diet-induced type 2 diabetes)	[43]
	Grinding machine	Sucrose density gradient with ultracentrifugation	DSS-induced colitis (0–50 mg/kg in C57BL/6J mice)	[44]
*Vitis vinifera* (grape)	Squeeze	Sucrose gradient	DSS-induced colitis (2 mg/200 µL/mouse in Lgr5-EGFP-IRES-CreERT2 and C57BL/6J mice daily for 7 days)	[45]
*Brassica oleracea* L. (broccoli)	Grinding machine	Sucrose gradient with ultracentrifugation	Loperamide-induced constipation (17.5 mg/kg/d in LOP-induced constipated mice)	[46]
*Citrus limon*	Squeeze	30% sucrose/D_2_O cushion with ultracentrifugation	Apoptosis in chronic myeloid leukemia (CML) tumor growth (0–20 µg/mL in A549, SW480 and LAMA-84 cells and 0–50 µg in NOD/SCID mice bearing CML xenograft tumors)	[47]
*Salvia miltiorrhiza*	Grinding machine	Sucrose density gradient with ultracentrifugation	Neovascularization in myocardial ischemia–reperfusion injury (0–100 µg/mL in HUVECs and 10 mg/kg in C57BL/6 mice)	[48]
*Asparagus cochinchinensis*	Squeeze	Sucrose density gradient with ultracentrifugation	Cytotoxicity against human hepatocellular carcinoma (HCC) cells (0–50 µg/mL in HepG2, Hep3B, SMMC-7721, and LO2 cells and 15 mg/mouse in BALB/c and BALB/c nude mice bearing HepG2 xenograft tumors)	[49]
*Momordica charantia*	Grinding machine	Sucrose density gradient with ultracentrifugation	Neuroprotective effects against ischemic brain injury (IBI) (0–800 µg/kg in SD rats)	[50]
*Lycium barbarum* L. (goji berry)	Grinding machine	Sucrose density gradient with ultracentrifugation	Dexamethasone-induced muscle atrophy (0–1 × 10^9^ particles/mL in C2C12 cells and 1 × 10^8^ particles/mouse in C57BL/6J mice daily for 14 days)	[51]
*Citrus limon* (lemon)	Squeeze	Sucrose density gradient with ultracentrifugation	Bile resistance enhancement (1 × 10^10^/mL in *Lactobacillus rhamnosus* GG and 5 × 10^9^/g in C57BL/6	[52]
*Panax notoginseng*	Grinding machine	Sucrose density gradient with ultracentrifugation	Ischemia–reperfusion injury (3 mg/kg in SD rats)	[53]
*Lycium barbarum* L.	Grinding machine	Sucrose density gradient with ultracentrifugation	Spinal cord injury repair	[54]

**Table 3:** Exosomes isolated by polymer-based precipitation.

Exosome Origin	Extraction Method	Isolation Method	Application	Reference
*Solanum nigrum* L.	Grinding machine	PEG 6000 with centrifugation	Anti-inflammatory activity (0–2.5 µg/mL in RAW 264.7 cells)	[55]
*Pachyrhizus erosus* (yam bean)	Grinding machine	PEG 6000	Anti-melanogenic effect in skin treatment (0–7.5 μg/mL concentration on melanocyte cell number of Zebrafish)	[56]
Ginger rhizomes	Grinding machine	PEG 6000 or ultracentrifugation	Anti-oxidant activity	[57]
*Carica papaya* L.	Grinding machine	PEG 6000	Anti-inflammatory activity (0–100 µg/mL in RAW 264.7 cells)	[58]
*Physalis minima* (golden cherry)	Grinding machine	PEG 6000	Treatment of photoaging (approximately 400 µg/mL as the IC50 value in the DPPH assay)	[59]
*Allium tuberosum*	Crush	Commercial exosome isolation kit	Neuroinflammation (0–20 µg/mL in BV-2 cells)	[60]
*Atractylodes lancea* rhizome	Hot water extract	Commercial exosome isolation kit	Melanogenesis (0–20 µg/mL in B16-F10 melanoma cells)	[61]
	Hot water extract	Commercial exosome isolation kit	Anti-inflammatory effect in murine microglial cells (0–20 µg/mL in BV-2 cells)	[62]
*Vaccinium ashei* (blueberry)	Grinding machine	PEG 8000	Nonalcoholic fatty liver disease (0–200 µg/mL in HepG2 cells and 0–100 mg/mouse in C57BL/6 mice)	[63]
*Physalis peruviana* (goldenberry)	Grinding machine	PEG 6000	Human dermal fibroblast regeneration and remodeling (0–7.5 µg/mL in HDF cells)	[64]
	Grinding machine	PEG 6000	Anti-inflammatory potential by regulating macrophage M1/M2 polarization (0–40 µg/mL in RAW 264.7 cells)	[65]

**Table 4:** Exosomes isolated by size-exclusion chromatography.

Exosome Origin	Extraction Method	Isolation Method	Application	Reference
*Vaccinium ashei* (blueberry)	Squeeze	Ultracentrifugation with 10,000 MWCO membrane	Anti-inflammatory effect in EA.hy926 cells (0–40 µg/mL in EA.hy926 cells)	[67]
*Platycodon grandiflorum*	Grinding machine	TFF system	Acetaminophen-induced hepatotoxicity (100 µg/mL in RAW 264.7 cells and 200 µL of 1 mg/mL in C57BL/6J mice)	[68]
*Sesamum indicum* L. (sesame leaves)	Grinding machine	100 kD ultrafiltration membrane with ultrapure column	An anti-inflammatory effect in LPS-treated RAW 264.7 cells (0–40 µg/mL in RAW 264.7 cells)	[69]

## Data Availability

The original contributions presented in the study are included in this article, and further inquiries can be directed to the corresponding authors.

## Abbreviations

The following abbreviations are used in this manuscript:

ELNs	Exosome-like nanovesicles
MVBs	Multivesicular bodies
PEG	Polyethylene glycol
MHC	Major histocompatibility complex
CML	Chronic myeloid leukemia
IBI	Ischemic brain injury
TFF	Tangential flow filtration
SR	Scavenger receptor
TNBC	Triple-negative breast cancer
